# Erratum: A protein-truncating R179X variant in *RNF186* confers protection against ulcerative colitis

**DOI:** 10.1038/ncomms12869

**Published:** 2016-09-13

**Authors:** Manuel A. Rivas, Daniel Graham, Patrick Sulem, Christine Stevens, A. Nicole Desch, Philippe Goyette, Daniel Gudbjartsson, Ingileif Jonsdottir, Unnur Thorsteinsdottir, Frauke Degenhardt, Sören Mucha, Mitja I. Kurki, Dalin Li, Mauro D'Amato, Vito Annese, Severine Vermeire, Rinse K. Weersma, Jonas Halfvarson, Paulina Paavola-Sakki, Maarit Lappalainen, Monkol Lek, Beryl Cummings, Taru Tukiainen, Talin Haritunians, Leena Halme, Lotta L. E. Koskinen, Ashwin N. Ananthakrishnan, Yang Luo, Graham A. Heap, Marijn C. Visschedijk, UK IBD Genetics Consortium, J. Barrett, K. de Lange, C. Edwards, A. Hart, C. Hawkey, L. Jostins, N. Kennedy, C. Lamb, J. Lee, C. Lees, J. Mansfield, C. Mathew, C. Mowatt, W. Newman, E. Nimmo, M. Parkes, M. Pollard, N. Prescott, J. Randall, D. Rice, J. Satsangi, A. Simmons, M. Tremelling, H. Uhlig, D. Wilson, NIDDK IBD Genetics Consortium, C. Abraham, J.P. Achkar, A. Bitton, G. Boucher, K. Croitoru, P. Fleshner, J. Glas, S. Kugathasan, J. V. Limbergen, R. Milgrom, D. Proctor, M. Regueiro, P. L. Schumm, Y. Sharma, J. M. Stempak, S. R. Targan, M. H. Wang, Daniel G. MacArthur, Benjamin M. Neale, Tariq Ahmad, Carl A. Anderson, Steven R. Brant, Richard H. Duerr, Mark S. Silverberg, Judy H. Cho, Aarno Palotie, Päivi Saavalainen, Kimmo Kontula, Martti Färkkilä, Dermot P. B. McGovern, Andre Franke, Kari Stefansson, John D. Rioux, Ramnik J. Xavier, Mark J. Daly

Nature Communications 7 Article number: 1234210.1038/ncomms12342 (2016); Published 08 09 2016; Updated 09 13 2016

The HTML version of this Article incorrectly listed the authors of the UK IBD Genetics Consortium and the NIDDK IBD Genetics Consortium individually in the main author list. This has now been corrected in the HTML; the PDF version of the paper was correct from the time of publication.

