# Supplementary material for: A protein-truncating R179X variant in RNF186 confers protection against ulcerative colitis
Source: Nat Commun. 2016 Aug 9;7:12342. doi: 10.1038/ncomms12342 (PMC4980482; doi:10.1038/ncomms12342)
Supplement: Supplementary Information — Supplementary Figures 1-8, Supplementary Tables 1-3, Supplementary Notes 1-3 and Supplementary References [file ncomms12342-s1.pdf]

Supplementary Figures

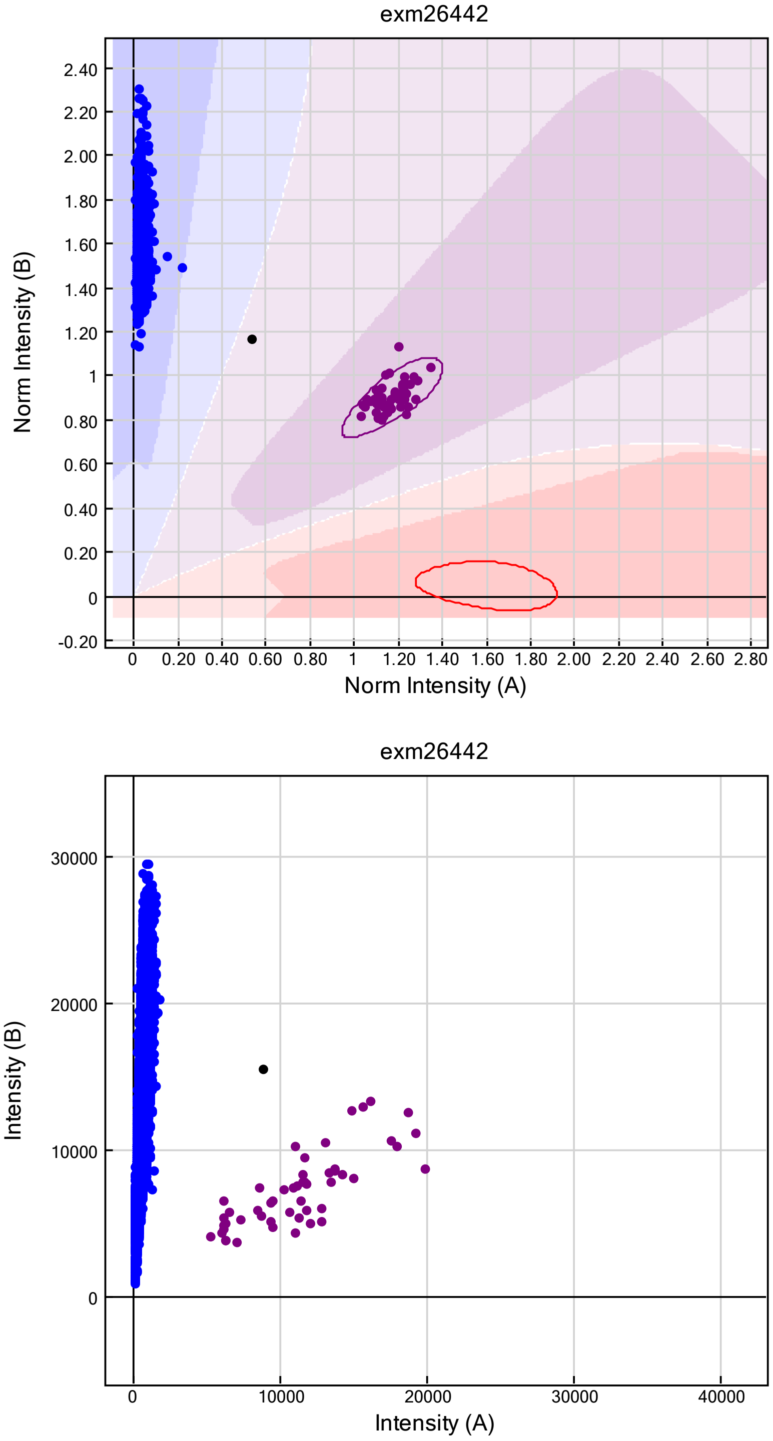

Supplementary figure 1. Cluster plots for rs36095412 (exm26442) from the Illumina HumanExome BeadChip array. Cartesian coordinates display the cluster using intensity values A and B representing the two possible alleles for this SNP. The top and bottom panels represent normalized and raw values, respectively. Red and blue shaded regions represent the two homozygous clusters; purple shaded region represents the heterozygous cluster.

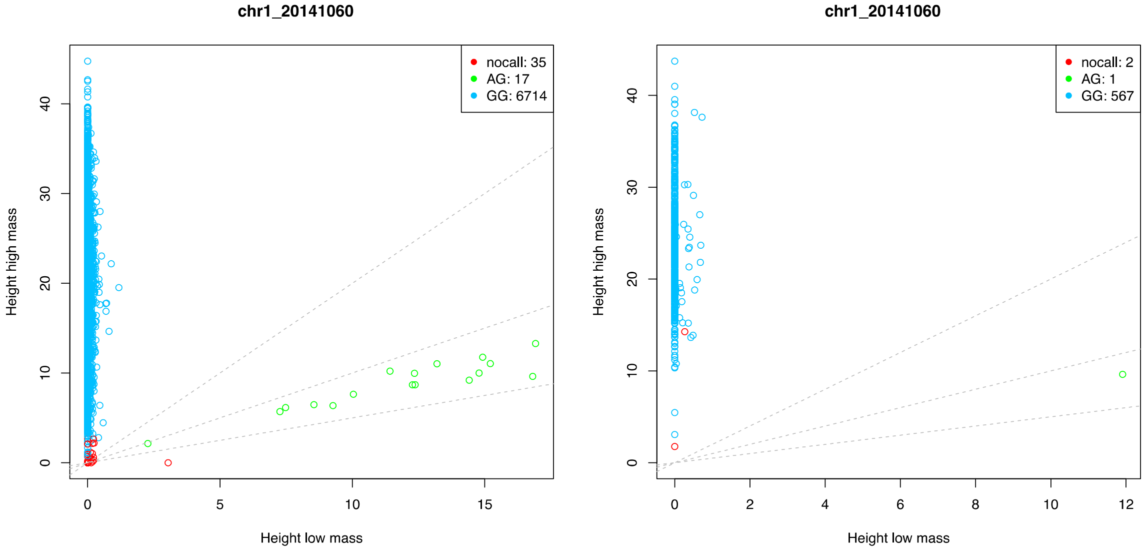

Supplementary figure 2. Cluster plots for rs36095412 (chr1_20141060) from the Sequenom genotyping. Using R and the raw data (heights of MASSSpec intensities): we obtained *skew*, where we divide allele with higher intensity by sum of height of both alleles; and *yield*, where we divide 1- height of unextended primer by sum of intensities of both alleles and unextended primer. *Yield* indicates quality of signal where a value below 0.5 indicates poor quality. *Height* refers to height of signal in the MassSpec.

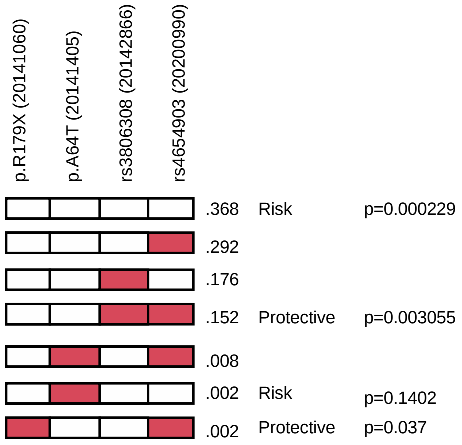

Supplementary figure 3. Haplotype blocks of RNF186 associated variants including: the low-frequency coding variants (1) p.R179X and (2) p.A64T; and the common associated variants (3) rs3806308 and (4) rs4654903. Haplotype-based case-control association analysis was conducted using PLINK 1.07 using a subset of individuals with array[1] and targeted genotyping data (red indicates the non-reference allele).

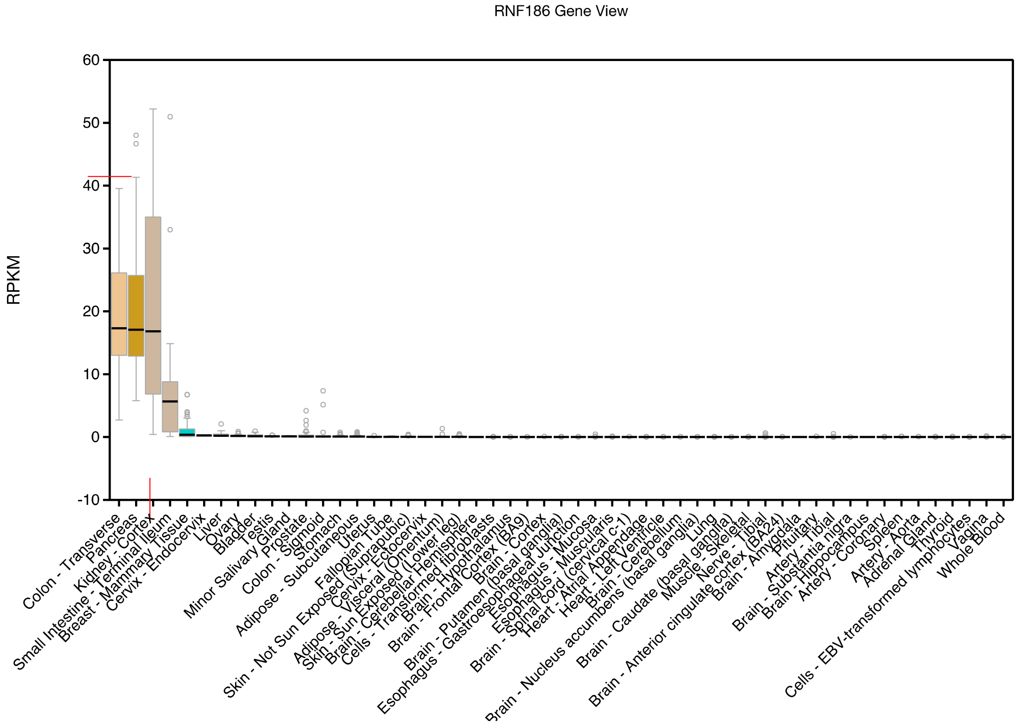

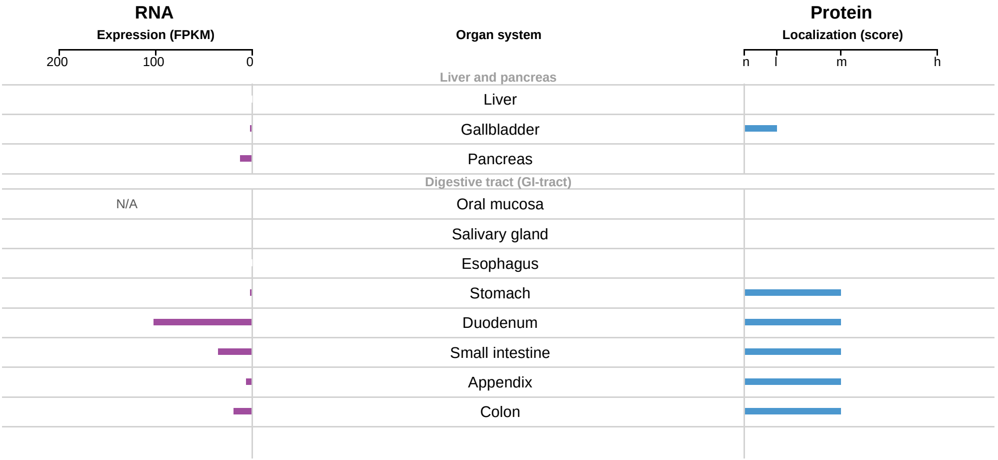

Supplementary figure 4. Tissue-wide RNA expression profile of *RNF186* in the Genotype-Tissue Expression[2] project (http://gtexportal.org/home/gene/RNF186). Tissues are sorted in decreasing order by the median RPKM value from GTEx Analysis Release V4. Protein expression profile of RNF186 protein in the human protein atlas[3] shows "medium" localization score in the digestive tract.

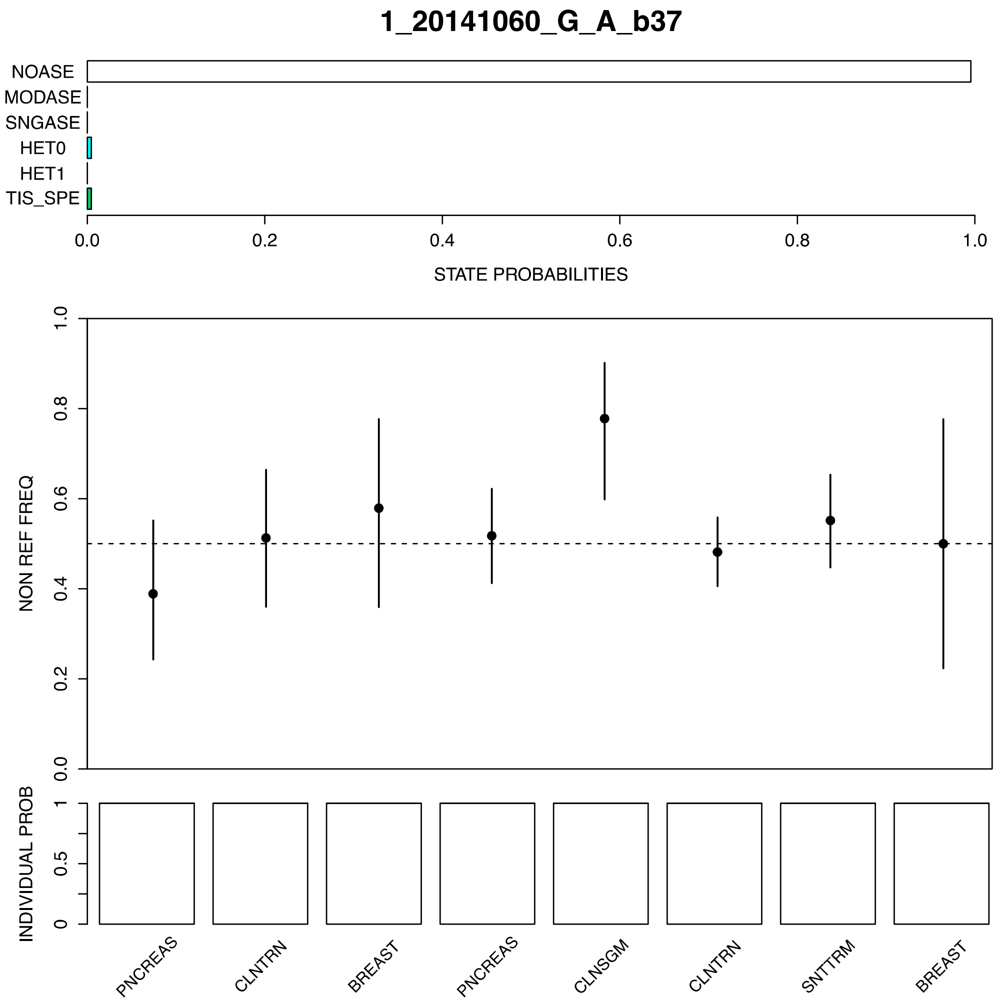

Supplementary figure 5. Allele-specific expression data for rs36095412 (p.R179X) in GTEx. Top panel shows the posterior probabilities for six states as defined in Pirinen et al[4]. The multi-tissue classification state ‘NOASE’ (no ASE effect across all tissues) has posterior probability greater than 0.9. Middle panel shows the point estimates of the non-reference allele frequency among RNA-seq reads across eight observations (in five different tissue types named at the bottom) together with their 95% credible intervals. Bottom panel shows the posterior probability of the group indication for each tissue type, where white, gray, and black denote groups no ASE, moderate ASE, and strong ASE, respectively.

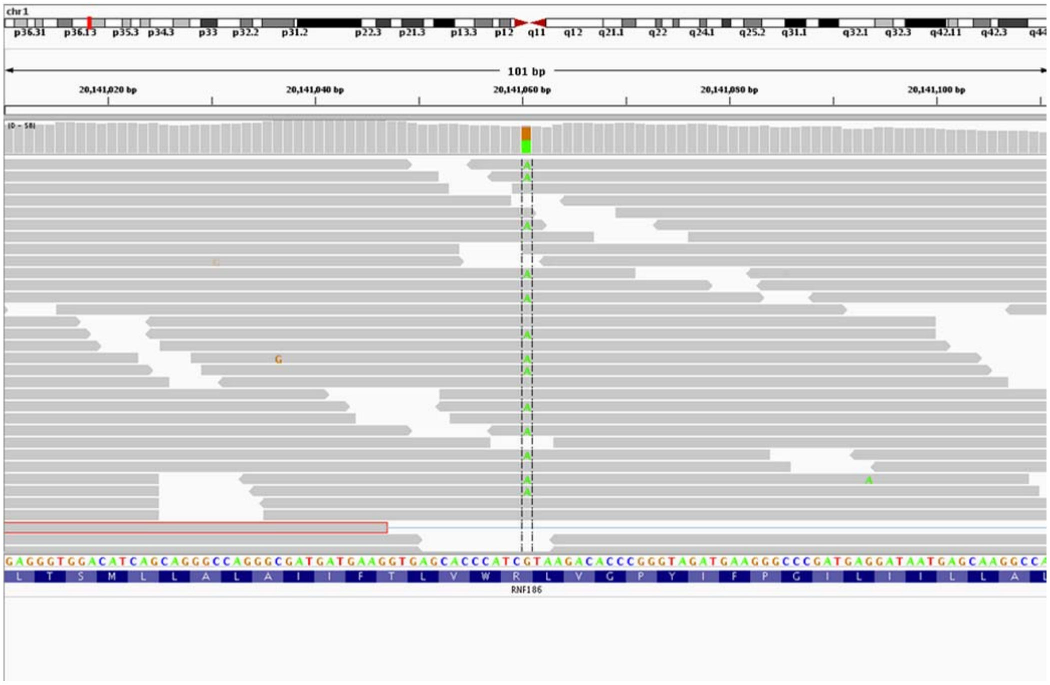

Colon transverse

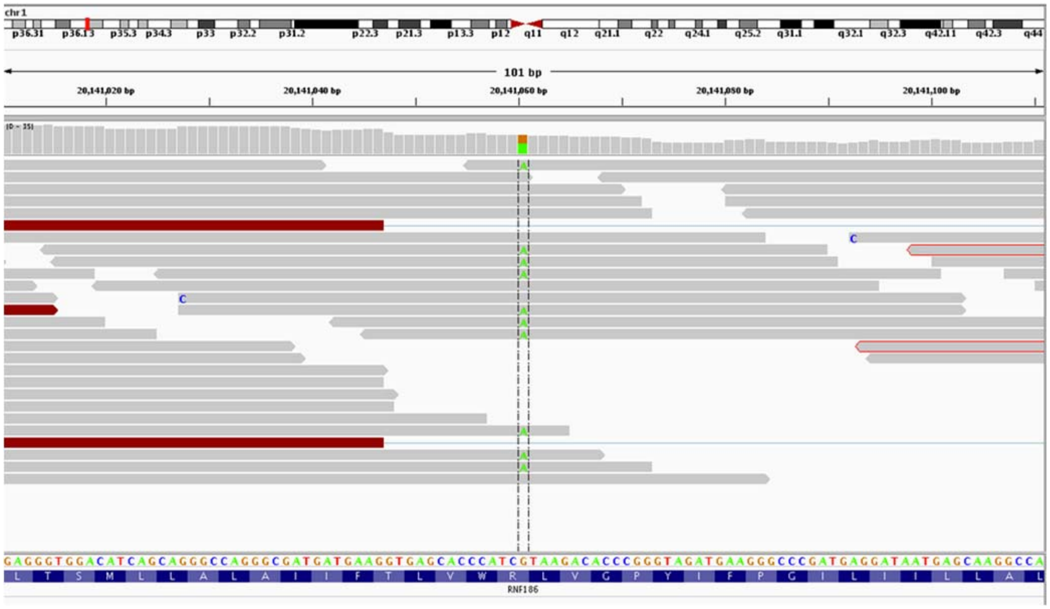

Breast mammary tissue

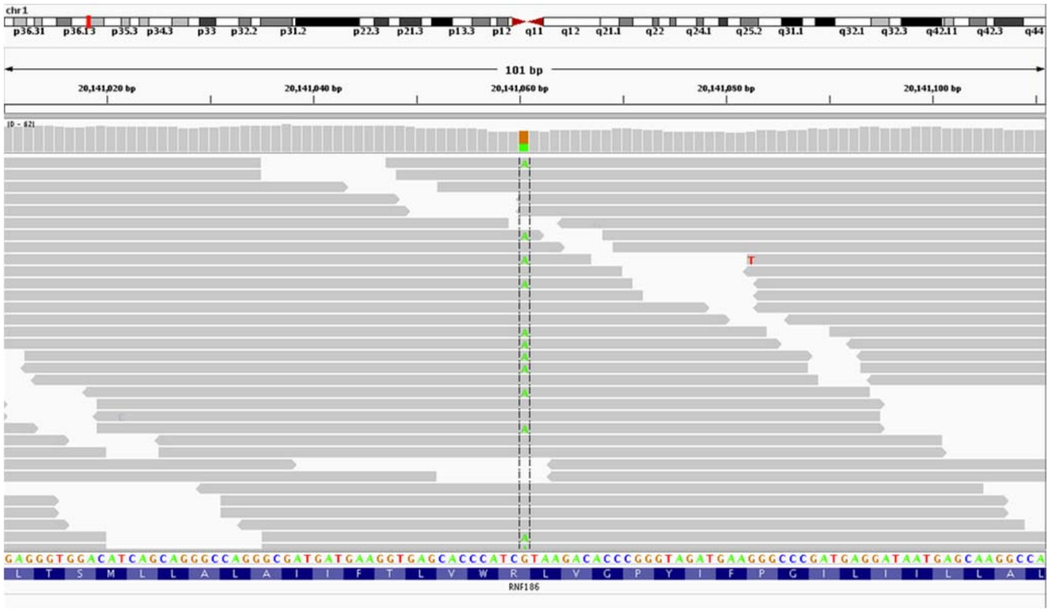

Pancreas

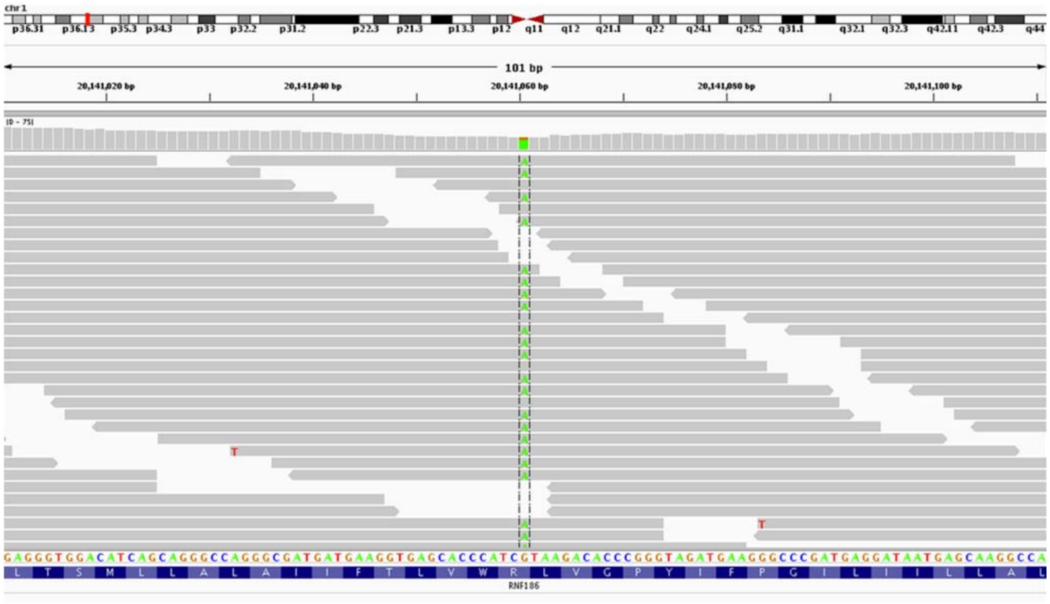

Colon sigmoid

Supplementary figure 6. IGV snapshot of RNA-seq reads from tissues with expression (RPKM) greater than zero in p.R179X carriers from the GTEx project.

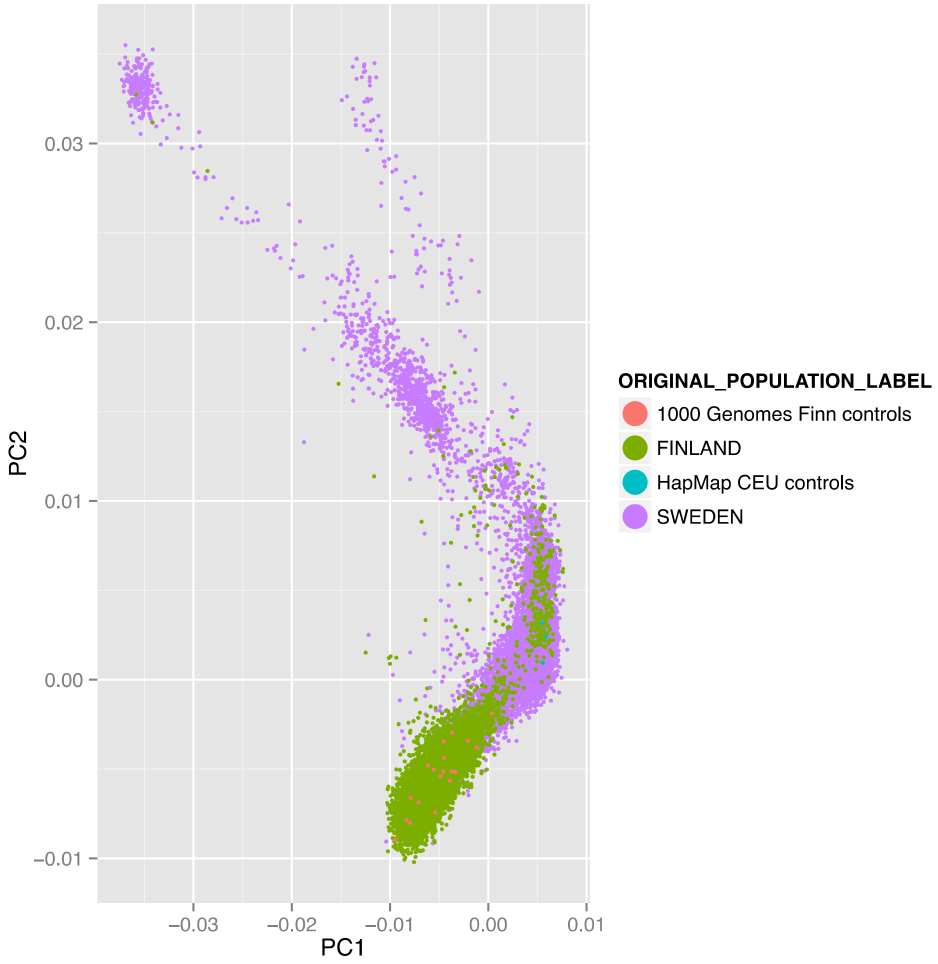

Supplementary figure 7. First two principal components showing genetic differences among 27885 jointly called individuals for classifying Finnish individuals among Swedish samples. Original population labels are given in different colors (Finnish, Swedish). Additionally, well-characterized control individuals were added to joint calling (Finnish individuals from 1000 genomes project and HapMap CEU individuals) and are shown in the figure as separate colors.

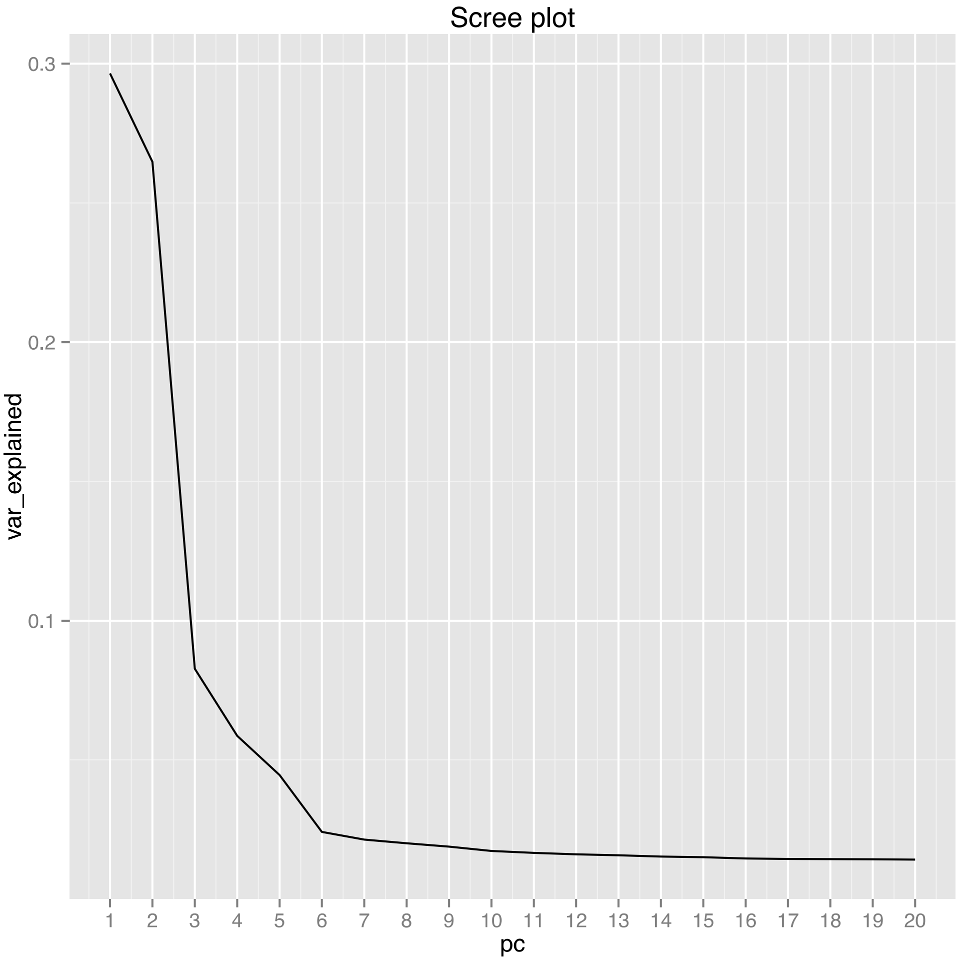

Supplementary figure 8. Proportion of genetic variance explained by first 20 principal components among 27885 sequenced individuals. In x-axis principal components are ordered by decreasing variance explained and y-axis gives variance explained by each principal component.

Supplementary Tables

Supplementary table 1. Prioritizing protective protein truncating variants identified in the targeted sequencing data set (CMH test). For each variant we present the analysis using the indexed association in Huang et al.[1] For each data set the single variant association analysis output from PLINK/SEQ is shown (https://atgu.mgh.harvard.edu/plinkseq/assoc.shtml#single). CONMETA – consensus sample-variant meta-information; ALT – alternate allele(s), comma delimited; MAF – minor allele frequency; HWE – *P*-value from Hardy-Weinberg disequilibrium test (exact test); MINA – number of minor alleles in cases; MINU – number of minor alleles in controls; OBSA – number of non-null genotypes in cases; OBSU – number of non-null genotypes in controls; REFA – number of reference homozygotes in cases; HETA – number of heterozygotes in cases; HOMA – number of alternate homozygotes in cases; REFU, HETU, HOMU – same as aforementioned description, but for controls; P – p-value for single site association (allelic, two-sided test); OR – Allelic odds ratio. Finnish exome data is also shown.

Supplementary table 2. Conditional analysis of p.R179X variant and the index common variant association rs4654903 reported in Silverberg et al (2009).[5] Conditional analysis results for the samples in the Iceland replication data set with whole genome sequencing data are shown.

	**rs36095412-A stop gained R179X (MAF 0.78%)**		**rs4654903-A Intergenic MAF(45.5%)**		**Phenotype (N)**
**Analysis**	**Pvalue**	**Effect**	**Pvalue**	**Effect**	
Unadjusted	5.0×10^{-4}	0.30	3.8×10^{-7}	1.24	Ulcerative Colitis (N Cases=1,453 ;Ctrls=264,744)
Adjusted for the other marker	8.4×10^{-4}	0.31	9.0×10^{-7}	1.23	

r^2 =0.004 ; D‘ = 0.83

Supplementary table 3. Association of p.R179X in *RNF186* with crohn’s disease

**Study**	**Data type**	**CD**		**Controls**		**Control MAF**	***P***	**OR**
		**179X**	**R179**	**179X**	**R179**			
GWASseq	Sequence (targeted)	4	2404	6	1828	0.33%		
Finland	Sequence (exome)	0	476	23	16223	0.14%		
**Screen**	-	4	2880	29	18051		0.19	0.36
US+Canada	Exome Chip	16	9962	21	12883	0.16%		
Sweden	Exome Chip	6	1108	45	10813	0.41%		
Belgium	Genotyping	3	3189	0	1764	0.00%		
Germany	Genotyping	4	2822	7	4399	0.16%		
Dutch	Genotyping	6	2306	8	4164	0.19%		
Italy	Genotyping	3	2249	2	1914	0.10%		
**Replication**							0.56	1.16 (0.76-1.76)
**Combined (screen +replication)**							0.94	1.04 (0.70-1.54)

CD, crohn's disease; OR, odds ratio; *P*, p-value. Screen + replication *P* value is computed using Mantel-Haenszel chi-squared test with continuity

Supplementary Notes

Supplementary Note 1: Pre-processing. The sequence reads are first mapped to the reference to produce a file in SAM/BAM format sorted by coordinate. Duplicate reads are marked – these reads are not informative and are not used as additional evidence for or against a putative variant. Next, local realignment is performed around indels. This identifies the most consistent placement of the reads relative to potential indels in order to clean up artifacts introduced in the original mapping step. Finally, base quality scores are recalibrated in order to produce more accurate per-base estimates of error emitted by the sequencing machines.

Supplementary Note 2: Variant Discovery. Once the data has been pre-processed as described above, it is put through the variant discovery process, i.e. the identification of sites where the data displays variation relative to the reference genome, and calculation of genotypes for each sample at that site. The variant discovery process is decomposed into separate steps: variant calling (performed per-sample), joint genotyping (performed per-cohort) and variant filtering (also performed per-cohort). The first two steps are designed to maximize sensitivity, while the filtering step aims to deliver a level of specificity that can be customized for each project.

Variant calling is done by running the HaplotypeCaller in GVCF mode on each sample's BAM file(s) to create single-sample gVCFs. If there are more than a few hundred samples, batches of ~200 gVCFs are merged hierarchically into a single gVCF to make the next step more tractable. Joint genotyping is then performed on the gVCFs of all available samples together in order to create a set of raw SNP and indel calls. Finally, variant recalibration is performed in order to assign a well-calibrated probability to each variant call in a raw call set, and to apply filters that produce a subset of calls with the desired balance of specificity and sensitivity.

Supplementary Note 3: Identification of Finnish samples. Initial data set consisted of 27885 jointly called individuals from Finnish and Swedish cohorts. GATK PASS SNPs were extracted that satisfied the following conditions: minor allele frequency > 0.05, HWE-p-value < 1e-6, missing genotypes <= 0.03 (after setting GQ<20 to missing). Remaining variants were LD pruned so that they were approximately independent (R2 < 0.1 within 500kb). Remaining SNPs were used for PCA-analysis.

Majority of the Finnish and Swedish samples clustered in to clear separate clusters based on PC1 and PC2 (Supplementary figure 7) as they explained over 50% of the variance after which there was a clear drop in variance explained (PC3 explained only 8%, Supplementary figure 8). For objectively classifying Finns we used 100 fold cross validation in logistic regression framework to estimate weights for the PC1 and PC2. In each round of cross validation PC1 and PC2 were regressed on population label and the Z-scores were stored. PC score was calculated by dividing the mean of the Z-scores for that PC by the standard deviation in cross validation replicated. Final weight was obtained by dividing a PC score by the sum of all PC scores. The weights were used to calculate weighted mahalanobis distance of each sample to the centroid of each population learning samples. Probability of sample coming from each

population was calculated by squaring the mahalanobis distance and getting cumulative density at that value from chisquare distribution with two degrees of freedom.

Supplementary References

1. Huang, H. *et al.* Association mapping of inflammatory bowel disease loci to single variant resolution. *bioRxiv* (2015).
2. Consortium, G.T. Human genomics. The Genotype-Tissue Expression (GTEx) pilot analysis: multitissue gene regulation in humans. *Science* **348**, 648-60 (2015).
3. Uhlen, M. *et al.* Proteomics. Tissue-based map of the human proteome. *Science* **347**, 1260419 (2015).
4. Pirinen, M. *et al.* Assessing allele-specific expression across multiple tissues from RNA-seq read data. *Bioinformatics* **31**, 2497-504 (2015).
5. Silverberg, M.S. *et al.* Ulcerative colitis-risk loci on chromosomes 1p36 and 12q15 found by genome-wide association study. *Nat Genet* **41**, 216-20 (2009).
